# Advertising

**Published:** 1888-06

**Authors:** 


### Editorial.

### ADVERTISING.

We have received a number of letters from esteemed correspondents, enclosing copies of newspaper cards and asking whether, in our opinion, they are contrary to the accepted code of dental ethics. In its proper sense, to advertise is to give notice, advice or intelligence; as "I will advertise thee what this people will do." (Num. xxiv, 14); and in this meaning advertising is entirely proper for dentists, or any professional man. But in its more modern sense, advertising is the extravagant laudation of merchandise, wares or commodities, which one has for sale. In this meaning, no respectable professional man can indulge in the practice. If he places his personal services on the same plane with the ready-made clothing of the merchant, to be sold to him who will pay the most, he is a huckster, and has no place with professional men, for his professional services are not reserved for those who are in distress, but are vended out to whomsoever can be prevailed upon to pay their price.

It is entirely proper for any professional man to advertise (apprise) people of a change of residence, for instance. He may, with propriety, inform them that his practice is confined to any particular specialty, or that he is prepared to perform unusual operations, such as oral surgery, or that he makes continuous gum-work, or obturators, or even that he keeps nitrous-oxide for administration. He may publicly present a card giving his office address and business, but he may not vaunt his skill, or claim superior ability, or boast of special and exclusive privileges. He cannot call attention to his unusually low prices, because these methods belong to trade, and not a profession. In short, it is the spirit which animates him and prompts his public notices that stamps his status. We have seen newspaper advertisements, occupying, perhaps, considerable space, which were not in contravention of the code of ethics, and we have seen three-line notices which were entirely unprofessional.

It is extremely difficult to reduce to a written code all the laws which should govern in professional matters. There is an unwritten law which is superior to all enactments, and which cannot be infringed without branding the man as unprofessional. One cannot continually depreciate his professional neighbors and disparage their operations, without subjecting himself to the penalties of the written code. But there is a way of doing this without actual words, by a shrug of the shoulders, a turn of the lip, a sniff or a sneer, that will not come within the written law, but which will, perhaps, in even a greater degree, be unprofessional. Not only this, but it will be infinitely meaner and more rascally vile and dishonorable than an open, sweeping condemnation in words. So a newspaper card may be so contemptible in its implications, without actually overstepping the written code, that it is more unprofessional than the braggart vaporings of a silly fool.

A man may offensively advertise without resort to newspapers or handbills. He may affect peculiarities of dress, or of conduct on the streets, for the purpose of calling public attention to himself. He may seek notoriety in many ways, that shall mark him as a charlatan. He may continually boast of his great achievements, or of his overwhelming practice, in a manner that writes him down a professional quack. We have heard the most offensive advertising speeches in dental society meetings, and that, too, from men who would be the first to call out for crucifixion of the dentist who should publish a card in the newspapers. We have read accounts in the dental journals of the unvarying success in the treatment of certain diseases, by men who had the hardihood to affix their names to the braggart advertisements of their professional skill—men who virtually claimed to be exempt from the common lot of mankind, and to be infallible and beyond mistakes. We have editorially struck out more than one such pitiful exhibition on the part of writers for this journal, for these are some of the most offensive forms of advertising.

We have heard of an obstetrician whose engagements extended a year in advance, and could not but wonder at the prescience exhibited by ——— somebody. But the braggart swaggering of this man has been exceeded by some of our modern dentists, who, if they may be believed, have their appointment books filled for a term almost equal to that of a natural life. Not long since we overheard a dentist, who has not the reputation of possessing anything more than a respectably good practice, who has no assistant and who does any kind of work that is offered, boasting that each week he rolled out from fifty to seventy-five dollars in coin for crown and bridge-work. A little calculation was all that was necessary to prove what an unconscionable liar and an offensive advertiser he was.

We might instance other ways in which dentists may unprofessionally advertise themselves. But the whole would be lost upon him who cannot see that the code of dental ethics is not an inflexible written law, by whose measured paragraphs the actions of men who claim professional status are to be judged. It is rather an indication, a sign, an intimation of that higher law, that elevated, pure, professional feeling which unerringly guides a man in professional ways, for he who prostitutes an honorable position to unworthy ends and purposes, who degrades a high calling to mere money-getting and sordid avariciousness, is not a professional man, no matter what may be his station or how correct his outward deportment. The true professional man is a gentleman, and in all his professional acts he will be prompted by gentlemanly instincts.

We might as well preach the sermon out, for the text, like charity, covers a multitude of sins. Dental journals sometimes, thoughtlessly we believe, encourage the advertising proclivity of dentists. One such proposes—for a consideration—to carry the name of any dentist who will pay the fee, for one year, in a conspicuous page especially devoted to this purpose. It is not difficult to imagine the class of dentists who will avail themselves of this delicate offer. Another publisher issues various editions of a so-called dental journal, and for a comparatively small sum will print a special edition of one of its issues, with the name of the patronizing dentist at the head as editor and publisher, a certain amount of the space to be devoted to advertising him personally, after the manner of the advertising sheets of dry goods and clothing merchants. Such a thing *might* be made respectable and useful, but it is not at all probable that the money will be paid and the offer taken advantage of by a very reputable class of dentists. To our great surprise, we find the excellent and reputable *Archives of Dentistry* exchanging advertisements and offering clubbing terms with this affair.

Sometimes a young dentist who does not immediately leap into a great practice, dazzled by the apparent success of some advertising practitioner, is tempted to throw reputation to the dogs and sacrifice the esteem of his reputable brethren for the money which he expects to gain by unprofessional practices. He gets the idea that all one has to do is to advertise widely and recklessly, and certain pecuniary reward will inevitably follow. Now the real truth is, it requires greater business tact and ability to succeed by advertising than without it. The man who can make money in a profession by advertising, certainly could do so without it. Of all those who adopt this method, but a small percentage succeeds. The ratio of failures among advertising dentists is much greater than among those who pursue a professional course, because all intelligent people comprehend the fact that an advertising professional man is an anomaly. They believe that there must be something radically wrong about him, or he would not be obliged to descend to methods which all recognize as disreputable. An advertising doctor, lawyer, preacher or dentist, is known to be at war with all respectable professional ideas, and his patrons as a class must be sought among the ignorant, the uncultured and the generally impecunious. Such men are usually regarded as quacks and shysters, and if they succeed it is in spite of, and not because of, these methods. We have personally known a number of promising and able men who, not willing to wait for that plant of slow growth, public confidence, have entered upon a career of advertising, placed a great gulf between themselves and their professional brethren, and too late found out that success is not attained by violent measures. The only secure way is, by continued study and constant self-culture properly to prepare themselves for the service of their fellow-men, and by faithful work and honest, upright professional conduct, to prove themselves worthy of the patronage which, to such, will as surely come as the day follows the night.

